# PurN Is Involved in Antibiotic Tolerance and Virulence in *Staphylococcus aureus*

**DOI:** 10.3390/antibiotics11121702

**Published:** 2022-11-25

**Authors:** Qi Peng, Lu Guo, Yu Dong, Tingrui Bao, Huiyuan Wang, Tao Xu, Ying Zhang, Jian Han

**Affiliations:** 1 Department of Pathogenic Biology, School of Basic Medical Sciences, Lanzhou University, No. 199, Donggang West Rd., Lanzhou 730000, China; 2 Department of Infectious Diseases, Shanghai Key Laboratory of Infectious Diseases and Biosafety Emergency Response, National Medical Center for Infectious Diseases, Huashan Hospital, State Key Laboratory of Genetic Engineering, School of Life Science, Fudan University, Shanghai 200040, China; 3 State Key Laboratory for the Diagnosis and Treatment of Infectious Diseases, The First Affiliated Hospital, Zhejiang University School of Medicine, 79 Qingchun Rd., Hangzhou 310003, China

**Keywords:** *Staphylococcus aureus*, *purN*, persister, virulence, purine metabolism

## Abstract

*Staphylococcus aureus* can cause chronic infections which are closely related to persister formation. Purine metabolism is involved in *S. aureus* persister formation, and *purN*, encoding phosphoribosylglycinamide formyltransferase, is an important gene in the purine metabolism process. In this study, we generated a Δ*purN* mutant of the *S. aureus* Newman strain and assessed its roles in antibiotic tolerance and virulence. The Δ*purN* in the late exponential phase had a significant defect in persistence to antibiotics. Complementation of the Δ*purN* restored its tolerance to different antibiotics. PurN significantly affected virulence gene expression, hemolytic ability, and biofilm formation in *S. aureus.* Moreover, the LD_50_ (3.28 × 10^10^ CFU/mL) of the Δ*purN* for BALB/c mice was significantly higher than that of the parental strain (2.81 × 10^9^ CFU/mL). Transcriptome analysis revealed that 58 genes that were involved in purine metabolism, alanine, aspartate, glutamate metabolism, and 2-oxocarboxylic acid metabolism, etc., were downregulated, while 24 genes involved in ABC transporter and transferase activity were upregulated in Δ*purN* vs. parental strain. Protein-protein interaction network showed that there was a close relationship between PurN and GltB, and SaeRS. The study demonstrated that PurN participates in the formation of the late exponential phase *S. aureus* persisters via GltB and regulates its virulence by activating the SaeRS two-component system.

## 1. Introduction

*Staphylococcus aureus* is a common pathogen and usually resides asymptomatically on the skin and mucous membranes of humans and animals [1]. *S. aureus* can synthesize and produce various virulence factors, such as fibronectin-, fibrinogen-, and immunoglobulin-cell wall binding proteins and capsular polysaccharides, pore-forming toxins, enterotoxins, toxic shock syndrome toxin-1 (TSST-1), exfoliative toxins, multiple tissue-damaging exoenzymes, etc. [2,3,4,5,6]. These virulence factors and the biofilm, which are established by attaching to medical implants and host tissues, are responsible for a variety of acute or chronic and relapsing suppurative infections such as impetigo, bacteremia, and endocarditis, pneumonia and empyema, osteomyelitis, infections of implanted devices, septic arthritis, etc. [7,8] and toxin-mediated diseases including scalded skin syndrome, food poisoning and toxic shock [6]. *S. aureus* has become a significant burden on the health care system and a major cause of nosocomial and community-acquired infections [8]. Due to the formation of persisters and the emerging resistance to antibiotics, the treatment of *S. aureus* infections, especially chronic and relapsing infections, has become quite challenging [9].

Persisters are a small subpopulation of bacterial cells in a genetically homogenous population that show tolerance to lethal doses of antibiotics without genetic mutations and present as phenotypic variants in a nongrowing dormant state [10]. Persister cells have been identified in every major pathogen [11,12], such as *Borrelia burgdorferi*, *Mycobacterium tuberculosis*, *S. aureus*, *Escherichia coli*, *Pseudomonas aeruginosa*, *Salmonella* typhimurium, etc. and are responsible for post-treatment relapse and can lead to chronic and recurrent infections [13,14,15,16,17,18].

Persisters are dormant cells [10,19]; however, there are similarities and differences in the mechanisms by which different bacteria form persisters. The mechanisms of persister formation and survival have been studied mainly in *E. coli*, and various genes and pathways have been confirmed to be involved in persister formation or survival [12]. The best-known pathways include toxin-antitoxin modules (HipA/B) [20]; energy production (SucB, UbiF) [21]; the trans-translation mediated pathway (SsrA and SmpB) [22]; the stringent response (RelA) [23]; the phosphate and cellular metabolism PhoU-mediated pathway [24]; SOS response/DNA repair (LexA) [25], etc. However, the mechanisms of persistence in *S. aureus* are not well understood. Recent studies have identified several pathways involved in persister formation in *S. aureus*, such as biosynthesis of amino acids (ArgJ) [26]; purine biosynthesis metabolism (PurF, PurB, and PurM) [27,28]; energy production (CtaB, SucA, SucB, SdhA, and SdhB) [29,30,31]; glycerol metabolism [32]; protein degradation (ClpX) [31]; and phosphate metabolism (PhoU) [33]. Numerous studies have demonstrated that persister formation in stationary phase bacteria is significantly higher than that of the bacteria in the exponential phase [10,12,34,35,36,37]. This indicates that there may be differences in the mechanisms of persister formation at different growth phases. Furthermore, multiple persistence-related genes such as *argJ*, *lysR*, *phoU*, and *msaABCR* [26,33,38,39] are involved in regulating *S. aureus* virulence, indicating that the persister formation mechanism is associated with virulence.

Previously, we found that purine metabolism plays a role in antibiotic tolerance and that PurB and PurM are involved in persister formation in *S. aureus* [27]. *purN*, encoding phosphoribosylglycinamide formyltransferase, is an important gene in the purine metabolism process. PurN catalyzes glycinamide ribonucleotide (GAR) to formylglycinamide ribonucleotide (fGAR), which is an important step to produce inosine monophosphate (IMP) [40]. In this study, we generated a purN mutant of the *S. aureus* Newman strain, and the effects of the *purN* deletion on bacterial growth, antibiotic tolerance, and virulence were investigated. Mutation analysis indicated that *purN* was important for persister formation and virulence in *S. aureus*. Our work provides new insights into the mechanisms of antibiotic tolerance and the factors affecting virulence in *S. aureus* and furnishes new therapeutic targets for improved treatment of *S. aureus* persistent infections.

## 2. Results

### 2.1. ΔpurN Had Significantly Decreased Antibiotic Tolerance

Based on our previous study, that PurB and PurM participated in purine metabolism and were involved in persister formation in *S. aureus* [27], we constructed a mutant strain of *purN* encoding phosphoribosylglycinamide formyltransferase in *S. aureus* Newman strain by homologous recombination to further explore the mechanisms by which purine metabolism regulates persister formation and virulence of *S. aureus* in this study.

**Figure 1:**
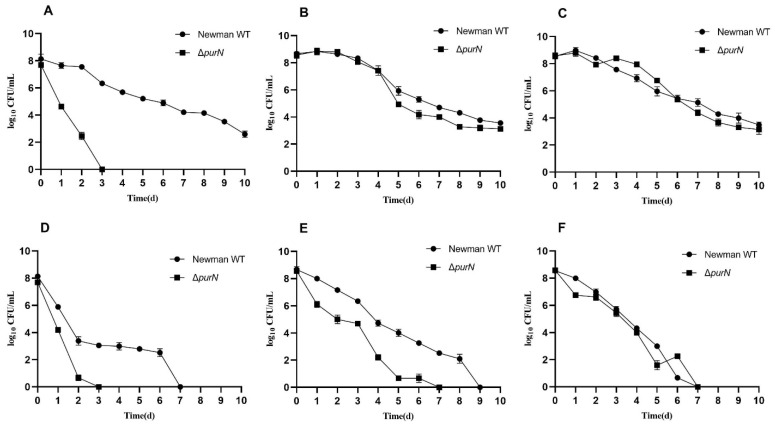
Exposure assay results of *S. aureus* wild-type, Δ*purN* to ampicillin (10 μg/mL, (**A**–**C**)), and levofloxacin (20 μg/mL, (**D**–**F**)) in cultures at different time points. 5 h point (**A**,**D**). 9 h point (**B**,**E**). 18 h point (**C**,**F**).

In order to investigate the effect of the *purN* knockout on the formation of *S. aureus* persisters, antibiotic exposure tests at different culture time points were performed to determine the survival of the wild-type and Δ*purN*. Compared to the parental strain, Δ*purN* showed significantly increased susceptibility to ampicillin in 5-h cultures and was completely killed after 3 days of drug exposure, while the wild-type had approximately 10^6^ CFU/ mL of viable cells remaining. Even on the 10th day of ampicillin treatment, the wild-type still had 10^2^ CFU/mL of bacteria remaining (Figure 1A). There were no significant differences in the survival of the wild-type and Δ*purN* strains upon ampicillin exposure when the bacteria were cultured for 9 and 18 h. Approximately 10^3^ CFU/mL of bacteria remained after 10 days of drug exposure (Figure 1B,C).

Similar results were observed for levofloxacin exposure. Compared with the parental strain, Δ*purN* showed increased sensitivity to levofloxacin when the bacteria were cultured for 5 and 9 h (Figure 1D,E). Among them, the most significant difference was observed in the 5-h cultures. After 3 days of levofloxacin exposure, Δ*purN* exhibited no surviving bacteria, whereas more than 10^3^ CFU/mL of bacteria remained for the parental strain. The persister level of *S. aureus* wild-type with levofloxacin exposure was similar to that of Δ*purN* in 18-h cultures (Figure 1F).

### 2.2. Complementation of the purN Restored Tolerance to Various Antibiotics

To further confirm the relationship between *purN* and *S. aureus* persister formation, the pRAB11 plasmid was used to complement ∆*purN* and the wild-type. Newman::pRAB11, ∆*purN*::pRAB11, ∆*purN*::pRABpurN, and Newman::pRBpurN were successfully obtained. The growth curves for these four strains indicated no differences in either the log phase or stationary phases under non-stressed conditions (Supplementary Figure S1). Compared with the Newman::pRAB11 strain, RT-qPCR confirmed that the expression levels of *purN* in the complemented ∆*purN*::pRABpurN strain (log_2_ fold change: 5.58 ± 0.16) and Newman::pRBpurN strain (log_2_ fold change: 6.48 ± 0.22) induced by anhydrotetracycline (Atc) were significantly higher than that of the wild-type with pRAB11 (*p* < 0.05).

**Figure 2:**
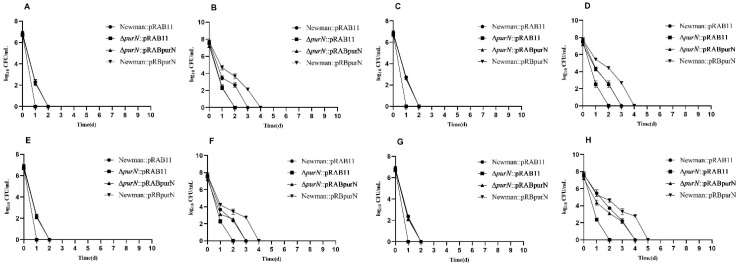
Drug exposure results of Newman::pRAB11, ∆*purN*::pRAB11, ∆*purN*::pRABpurN, and Newman::pRBpurN to ampicillin (**A**,**B**), vancomycin (**C**,**D**), gentamicin (**E**,**F**) and levofloxacin (**G**,**H**) at different culture times. 5-h culture (**A**,**C**,**E**,**G**); 9-h culture (**B**,**D**,**F**,**H**).

An antibiotic exposure experiment was carried out for the constructed *S. aureus* strains. Due to the pRAB11 used in the complementation study being an Atc induced plasmid, all the complemented strains were cultured in TSB medium containing Atc (100 ng/mL) which can produce certain inhibition of *S. aureus* growth. The growth rates of each *S. aureus* complemented by pRAB11 or pRABpurN significantly decreased so that the numbers of live bacteria were still less than 10^8^ CFU/mL after 9 h of culture, and they were still in the exponential phase. In 5-h culture, the antibiotics (e.g., ampicillin, vancomycin, gentamicin, and levofloxacin) exposure experiment demonstrated that ∆*purN*::pRAB11 all died after 24 h of drug treatment, while Newman::pRAB11, ∆*purN*::pRABpurN, and Newman::pRBpurN had more than 10^2^ CFU/mL of bacteria remaining. After 48 h of antibiotic exposure, the Newman::pRAB11, ∆*purN*::pRABpurN, and Newman::pRBpurN strains had no viable bacteria (Figure 2A,C,E,G). Similar growth curves were observed in the 9-h cultures (Figure 2B,D,F,H). The *purN* complemented strain restored tolerance to antibiotics (e.g., vancomycin, gentamicin, and levofloxacin) except for ampicillin. However, for the 18-h cultures, except for the ∆*purN*::pRAB11, which had less than 10^3^ CFU/mL of bacteria remaining after 10 days of levofloxacin exposure, the other strains showed significant tolerance to ampicillin, vancomycin, gentamicin, and levofloxacin, with many viable bacteria remaining after 10 days of antibiotic exposure (Supplementary Figure S2A–D).

### 2.3. Knockout of purN Affected the Expression of Virulence Factors in S. aureus

**Figure 3:**
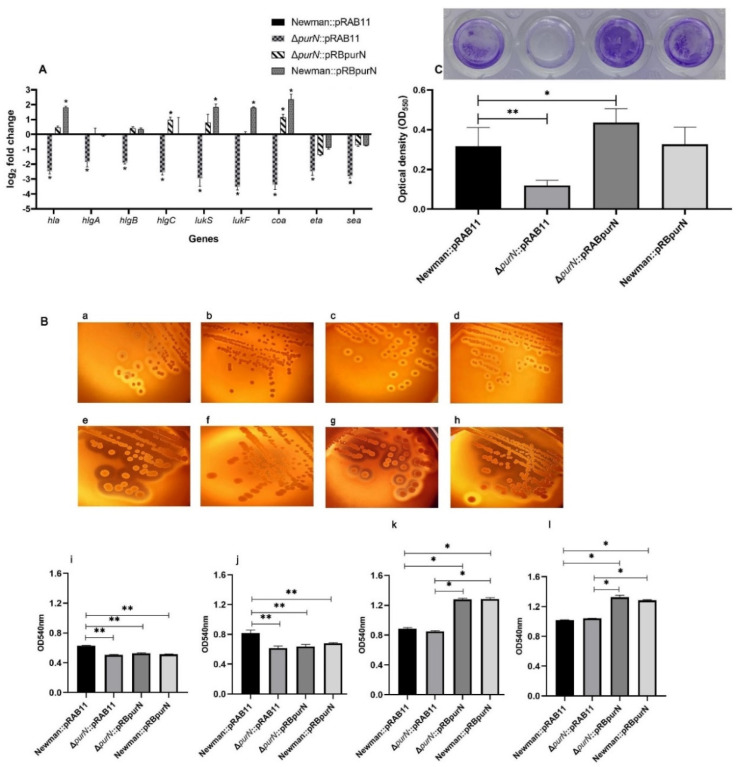
Comparison of the virulence of Newman::pRAB11, ∆*purN*::pRAB11, ∆*purN*::pRABpurN, and Newman::pRBpurN in *S. aureus*. (**A**) The virulence gene expression levels detected by RT-qPCR. (**B**) Variation of hemolysis in different strains. Hemolysis status of Newman::pRAB11 (**a**,**e**), ∆*purN*::pRAB11 (**b**,**f**), ∆*purN*::pRABpurN (**c**,**g**), and Newman::pRBpurN (**d**,**h**) cultured for 24 h (**a**–**d**) and 48 h (**e**–**h**) on blood TSA plates. The hemolysis assay of the four strains was measured in different time points cultures. (**i**)10 h, (**j**) 14 h, (**k**) 24 h, and (**l**) 48 h. (**C**) The biofilm formation abilities of the four *S. aureus* strains in 96-well plates. Comparison of OD_550_ and biofilm images in 96-well plate of different strains. * *p* < 0.05, ** *p* < 0.01.

To further investigate the effect of *purN* knockout on the expression of *S. aureus* virulence factors, RT-qPCR was used to compare the gene expression levels of the major virulence factors, including *hla*, *hlgA*, *hlgB*, *hlgC*, *lukS*, *lukF*, *eta*, *sea*, and *coa*, in the *S. aureus* Newman::pRAB11, Δ*purN*::pRAB11, Δ*purN*::pRBpurN, and Newman::pRBpurN strains. The expression levels of the major virulence genes in the Δ*purN*::pRAB11 strain were significantly lower than those in the Newman::pRAB11 strain (*p* < 0.05). The complemented strain, Δ*purN*::pRBpurN, exhibited restored expression levels of virulence genes. Moreover, the expression levels of *hlgC* and *coa* in Δ*purN*::pRBpurN were significantly higher than those for Newman::pRAB11 (*p* < 0.05). In addition, the expression levels of *hla*, *lukS*, *lukF,* and *coa* in Newman::pRBpurN were significantly higher than those in Newman::pRAB11 (*p* < 0.05) (Figure 3A).

### 2.4. The Ability of the ∆purN to Lyse Sheep Erythrocytes Was Significantly Reduced

To investigate the effect of the *purN* mutation on the hemolysis characteristics of *S. aureus*, the Newman::pRAB11, Δ*purN*::pRAB11, Δ*purN*::pRBpurN, and Newman::pRBpurN strains were inoculated on sheep blood TSA plates containing Atc (100 ng/mL) at 37 °C for 10, 14 (images not shown), 24 and 48 h, respectively. The β-hemolytic rings around the colony of the Newman::pRAB11 colony (Figure 3(Ba,Be)) were larger and clearer than those of Δ*purN*::pRAB11 (Figure 3(Bb,Bf)) at 24 and 48 h. In the 24- and 48-h cultures, the hemolytic rings of Δ*purN*::pRBpurN (Figure 3(Bc,Bg)) and Newman::pRBpurN (Figure 3(Bd,Bh)) tended to be consistent with that of Newman::pRAB11. Hemolysis assays of each *S. aureus* culture indicated that at 10 and 14 h, the hemolyzing ability of Newman::pRAB11 cultures was significantly higher than that for Δ*purN*::pRAB11 (*p* < 0.01, Figure 3(Bi,Bj)). With the prolongation of culture time and accumulation of hemolytic toxins, the differences in hemolytic ability between Newman::pRAB11 and Δ*purN*::pRAB11 disappeared at 24 h and 48 h. However, when purN was overexpressed, compared with Newman::pRAB11 and Δ*purN*::pRAB11, the hemolytic ability of the Δ*purN*::pRBpurN and Newman::pRBpurN strains was enhanced (*p* < 0.05, Figure 3(Bk,Bl)).

### 2.5. Knockout of purN Affected Biofilm Formation in S. aureus

The biofilm formation abilities of Newman::pRAB11, Δ*purN*::pRAB11, ∆*purN*::pRABpurN, and Newman::pRBpurN were measured in 96-well plates. The results showed that the biofilm formation ability of Newman::pRAB11 was significantly higher than that of Δ*purN*::pRAB11 (*p* < 0.01, Figure 3C). After complementation, the biofilm formation ability of Δ*purN*::pRBpurN was restored. Furthermore, when *purN* was overexpressed, the biofilm formation ability of Δ*purN*::pRBpurN was significantly higher than that of Newman::pRAB11 (*p* < 0.05, Figure 3C). In addition, there were no significant differences in biofilm formation between Newman::pRAB11 and Newman::pRBpurN.

### 2.6. The LD_50_ Values of ΔpurN in Mice Were Significantly Higher Than That of Wild-Type S. aureus

To further explore the effect of the purN mutation on the virulence of *S. aureus*, we determined the LD_50_ of the *S. aureus* Newman strain and the Δ*purN* in BALB/c mice. Different doses of the wild-type and Δ*purN* bacterial suspensions were injected intraperitoneally. The LD_50_ values for the wild-type and Δ*purN* in BALB/c mice were calculated according to the survival status of the mice, and the results showed that the LD_50_ of the Δ*purN* mutant (3.28 × 10^10^ CFU/mL) was significantly higher than that of the wild-type (2.81 × 10^9^ CFU/mL).

### 2.7. Comparative Transcriptome Analysis of the ΔpurN and the Wild-Type

**Figure 4:**
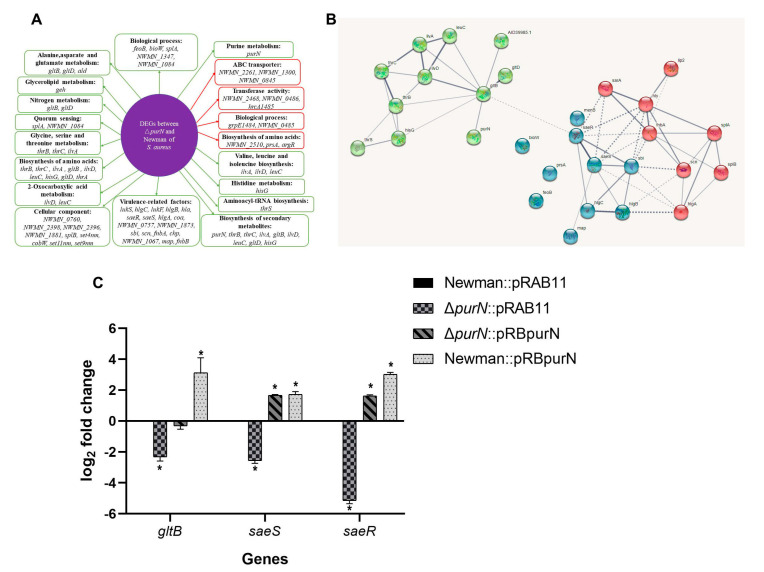
Comparative analyses of the transcriptomics of Δ*purN* and wild-type, and the *gltB*, *saeR*, and *saeS* expression in Newman::pRAB11, Δ*purN*::pRAB11, Δ*purN*::pRBpurN, and Newman::pRBpurN strains. (**A**) DEGs and pathways involved in the comparison of Δ*purN* and wild-type. The genes in the green box and red box are downregulated and upregulated genes, respectively. (**B**) Protein-protein interaction network of DEGs between Δ*purN* and parental strain by STRING database. The line thickness of the network indicates the strength of association/binding. (**C**) Comparison of expression levels of the *gltB*, *saeR*, and *saeS* in the four *S. aureus* strains (* *p* < 0.05).

To gain further insights into the molecular mechanisms by which PurN affects persister formation and virulence in *S. aureus*, the DEGs of the Δ*purN* mutant and the wild-type strain were profiled by RNA-seq. Compared with its parental strain, 58 genes were downregulated, and 24 genes were upregulated in the Δ*purN* mutant with a cutoff value of log_2_ fold change less than −2 or more than 2 (Supplementary Table S1). Thirteen DEGs were selected as target genes (e.g., *saeS*, *saeR*, *ilvA*, *NWMN_1873*, *lukS*, *hla*, *hlgC*, *lukF*, *NWMN_2510*, *NWMN_2262*, *NWMN_2266*, *NWMN_0485*, and *NWMN_0845*) for validation by RT-qPCR and the results confirmed the reliability of the transcriptome analysis (Supplementary Table S2). The DEGs were assigned to the following functional categories. KEGG pathway enrichment analysis suggested that these DEGs were mainly involved in purine metabolism, alanine, aspartate, and glutamate metabolism, 2-oxocarboxylic acid metabolism, histidine metabolism, biosynthesis of amino acids, ABC transporters, quorum sensing, etc. (Figure 4A). To evaluate the DEG associations, a PPI was constructed based on the STRING database, and the network showed that there were close relationships between *purN* and *gltB* and *saeR* and *saeS* (Figure 4B). Furthermore, compared with the wild-type, the transcription levels of virulence-related genes, including *lukS*, *lukF*, *hlgA*, *hlgB*, *hlgC,* and *hla*, were downregulated significantly in the Δ*purN* mutant (Supplementary Table S1).

To further explore the relationships between *purN* and *gltB* and *saeR* and *saeS*, RT-qPCR was used to detect the *gltB, saeR,* and *saeS* expression in the Newman::pRAB11, Δ*purN*::pRAB11, Δ*purN*::pRBpurN, and Newman::pRBpurN strains. Compared with Newman::pRAB11, the expression level of *gltB* in Δ*purN*::pRAB11 was significantly lower (*p* < 0.05), whereas in Newman::pRBpurN, it was higher (*p* < 0.05), and there was no significant difference in Δ*purN*::pRBpurN. Meanwhile, compared with Newman::pRAB11, the expression levels of *saeR* and *saeS* in Δ*purN*::pRAB11 were significantly lower (*p* < 0.05), whereas in *purN* overexpressed strains, Δ*purN*::pRBpurN and Newman::pRBpurN were significantly higher (*p* < 0.05) (Figure 4C). *purN* affected the expression of *gltB, saeR,* and *saeS* in *S. aureus* and was consistent with the PPI network (Figure 4B).

### 2.8. purN Affects the Persister Formation in S. aureus via gltB

**Figure 5:**
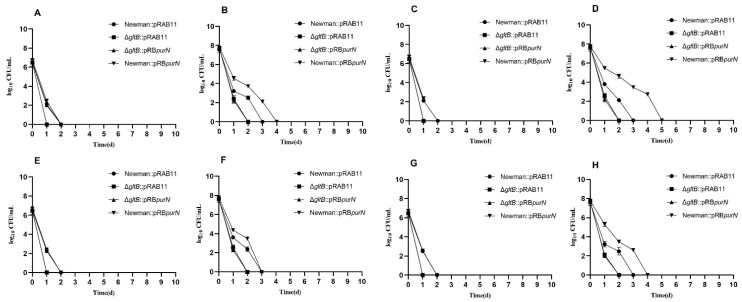
Antibiotics exposure results of Newman::pRAB11, ∆*gltB*::pRAB11, Δ*gltB*::pRBpurN and Newman::pRBpurN to ampicillin (**A**,**B**), levofloxacin (**C**,**D**), gentamicin (**E**,**F**) and vancomycin (**G**,**H**) at different culture times. 5-h culture (**A**,**C**,**E**,**G**); 9-h culture (**B**,**D**,**F**,**H**).

To verify the PPI network based on the transcriptome analysis of the Δ*purN* mutant and the wild-type, Δ*gltB*::pRAB11 and Δ*gltB*::pRBpurN were constructed. Further experiments showed that Δ*gltB*::pRAB11, Δ*gltB*::pRBpurN, Newman::pRBpurN, and ∆*purN*::pRABpurN had similar growth curves (Supplementary Figure S1). RT-qPCR confirmed that the expression level of *purN* in the ∆*gltB*::pRABpurN strain (log_2_fold change: 4.99 ± 0.016) was significantly higher than that in Δ*gltB*::pRAB11 (*p* < 0.05). To further explore the association between *purN* and *gltB* in the formation of *S. aureus* persisters, four strains, Newman::pRAB11, Δ*gltB*::pRAB11, Δ*gltB*::pRBpurN, and Newman::pRBpurN, were incubated for 5, 9, and 18 h, respectively. Each strain was exposed to lethal concentrations of antibiotics, including ampicillin (10 μg/mL), levofloxacin (20 μg/mL), vancomycin (40 μg/mL), and gentamicin (100 μg/mL), to observe the differences in persister formation ability. The results showed that the four strains in 5-h cultures were completely killed after 1–2 days of drug exposure (Figure 5A,C,E,G). However, after 9 h of incubation, the changing characteristics of the viable in Δ*gltB*::pRBpurN and Δ*gltB*::pRAB11 strains were similar and were completely killed after 2 days of antibiotic exposure, while the Newman::pRAB11 and Newman::pRBpurN strains retained more than 10^2^ CFU/mL of viable bacteria. The Newman::pRAB11 was killed after 3 days of drug exposure, while Newman::pRBpurN was completely killed after approximately 4–5 days of antibiotic exposure (Figure 5B,D,F,H). In the 18-h cultures, the numbers of viable bacteria in Δ*gltB*::pRAB11 and Δ*gltB*::pRBpurN were less than those of Newman::pRAB11 and Newman::pRBpurN after 10 days of drug exposure (Supplementary Figure S3A–D). The results showed that when *gltB* was knocked out, overexpression of *purN* did not increase persister formation, indicating that *purN* affects persister formation in *S. aureus* via *gltB.*

## 3. Discussion

Persister formation in *S. aureus* is closely related to the growth phase of culture [10,12,35,36]. Previous studies have shown that purine biosynthesis plays an important role in persister formation in *S. aureus* [27]. *purN* is a crucial gene in the third step of purine biosynthesis. We analyzed the effect of the *purN* mutant of *S. aureus* and found that its mutation resulted in persister reduction in late exponential phase cultures, indicating PurN is important for persister formation.

*purN* participates in several important pathways, including purine metabolism, one carbon pool by folate, metabolic pathways, and biosynthesis of secondary metabolites. A large number of studies have confirmed that ATP production [16,41], alarmone ppGpp [10], amino acid synthesis, and metabolism in bacterial cells play important roles in the formation and regulation of persisters in bacteria [26,42,43].

**Figure 6:**
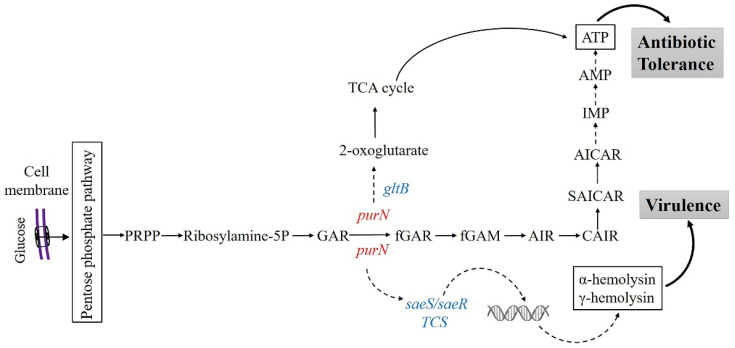
Pathways that indicate how *purN* is involved in persister formation and virulence in *S. aureus*.

It is well known that purine metabolism is crucial for ATP energy supply. PurN catalyzes GAR to fGAR, which is an important step in the purine metabolism process to produce IMP. IMP is converted to guanosine 5′-monophosphate (GMP) or adenosine 5′-monophosphate (AMP) by subsequent enzymes. In this process, both ribosylamine-5P produced by phosphoribosyl pyrophosphate (PRPP) and formylglycinamidine ribonucleotide (fGAM) produced by fGAR require glutamine to provide amido, and glutamate is also produced. At the same time, glycine is required to participate in the process of ribosylamine-5P to generate GAR, and aspartate is required to participate in the process of 5-amino-4-carboxyaminoimidazole ribonucleotide (CAIR) to generate N-succinylo-5-aminoimidazole-4-carboxamide ribonucleotide (SAICAR) [40] (Figure 6). The PPI network established by our data indicated that *purN* affected the persister formation in *S. aureus* via *gltB* (Figure 4B). *gltB,* encoding the large subunit of glutamate synthase, is the key gene in glutamine and glutamate metabolism, which catalyzes L-glutamine and 2-oxoglutarate into two molecules of L-glutamate [44]. Transcriptome analysis found that *gltB* expression decreased in the Δ*purN* mutant (Figure 4A). Thus, glutamine and glutamate synthesis were reduced. The decrease of *gltB* resulted in an increase of 2-oxoglutartate, which has been shown to promote the TCA cycle and cause increased ATP production [45,46], which in turn would inhibit persister formation of the Δ*purN* mutant (Figure 6).

Biofilm formation, a major virulence factor in *S. aureus* infections, accelerates bacterial colonization in host tissues and promotes persister formation and antimicrobial agents. Our data revealed that the *purN* mutant significantly decreased biofilm formation. In other previous studies, purine biosynthesis was shown to affect biofilm formation through the secondary messenger, cyclic di-AMP (c-di-AMP) [28,47]. PurN is involved in the third step of purine biosynthesis, which affects ATP production. c-di-AMP is synthesized by di-adenylate cyclase via the condensation of two ATPs, one of the final products of purine biosynthesis [48]. The Δ*purN* mutant may inhibit c-di-AMP synthesis from preventing bacterial biofilm formation in *S. aureus.* However, the underlying mechanisms deserve future detailed studies.

*S. aureus* has a complex regulatory network to control its virulence [49]. The regulatory systems include the accessory gene regulator (*agr*) quorum-sensing system [50], SarA protein family regulators [51], two-component system (TCS) of the SaeRS [52], SrrAB [53], ArlRS [54], and the alternative sigma factors (SigB and SigH) [51]. Transcriptome analyses of Δ*purN* and wild-type strains indicate that the expression levels of *saeR* and *saeS* encoding the SaeRS TCS were significantly decreased in the Δ*purN*, and due to this, the expression levels of multiple virulence factors, including α-hemolysin, γ-hemolysin, PVL, and coagulase, were also significantly reduced. This is consistent with our mouse study, in which we found that the virulence of Δ*purN* was significantly reduced, as well as the results of the hemolysis assay (Figure 3B). The SaeRS TCS is an important regulatory system for the virulence of *S. aureus* [52]. SaeS is the sensor histidine kinase, which can sense signals in the environment and autophosphorylate at the His131 residue and then the phosphoryl group is transferred to Asp51 of SaeR, and the phosphorylated SaeR (SaeR-P) binds to the SaeR binding sequence (SBS) to activate the transcription of the target genes [52,55,56]. Several Sae target genes have been discovered, most of which are related to the virulence of *S. aureus*, including *coa*, *fnbA*, *eap*, *sbi*, *efb*, *fib*, *saeP*, *hla*, *hlb*, and *hlgC* [57,58]. The currently reported signals of SaeRS TCS activation mainly include human neutrophil peptide 1, 2, and 3 (HNP1–3), calprotectin, hydrogen peroxide, etc. [59,60]. Our data showed that the expression levels of *saeR* and *saeS* were higher in the *purN* overexpressed strains (Figure 4C). The results confirmed the PPI networks (Figure 4B), which PurN may affect virulence through the SaeRS in *S. aureus* (Figure 6).

Our findings further suggest that there is a close relationship between persister formation and bacterial virulence. In addition to the reported multiple persistence-related genes, such as *argJ*, *lysR*, *phoU*, and *msaABCR*, which are involved in bacterial virulence [26,33,38,39], the PurN of *S. aureus* is another multifunctional factor that not only participates in persister formation but also participates in virulence regulation.

In summary, this study has demonstrated that PurN participates in the formation of the late exponential phase *S. aureus* persister formation via the key gene, *gltB,* in glutamate synthesis and regulates bacterial virulence by activating the SaeRS two-component system. Therefore, PurN can potentially serve as a novel therapeutic target to develop more effective treatments to control persistent *S. aureus* infections in the future.

## 4. Materials and Methods

### 4.1. Culture Media, Antibiotics, and Animals

Tryptic soy broth (TSB) and tryptic soy agar (TSA) were obtained from Becton Dickinson (BD). Luria-Bertani (LB) medium and anhydrotetracycline (Atc) were obtained from Solarbio (Beijing, China). The rationale for selecting the antibiotics used in antibiotics exposure experiments is based on clinically used antibiotics in treating *S. aureus* infections and three classes of bactericidal antibiotics commonly used for persister assays, i.e., cell wall inhibitors, aminoglycosides, and fluoroquinolones. Ampicillin, levofloxacin, rifampin, chloramphenicol, vancomycin, and gentamicin were obtained from Sangon Biotech (Shanghai, China), and their stock solutions were freshly prepared, filter-sterilized, and used at appropriate concentrations as indicated. BALB/c mice were purchased from Lanzhou University (China). The study was approved by the Ethics Committee of Lanzhou University.

### 4.2. Bacterial Strains and Culture Conditions

The bacterial strains and plasmids used in this study are listed in Table 1. All the *S. aureus* strains were cultivated in TSA and TSB. The *E. coli* DC10B strain was cultivated in LB. The shuttle vector, pRAB11, harbors a *tet* operator that is induced by Atc. In the process of inducing high expression of *purN*, *S. aureus* ∆*purN*::pRBpurN, Newman::pRABpurN, and ∆*gltB*::pRBpurN mutants, and the control strains, *S. aureus* ∆*purN*::pRB11, Newman::pRAB11, and ∆*gltB*::pRAB11 were all inoculated in TSB medium containing Atc (100 ng/mL). For the persister assays, antibiotics were used at the following concentrations: ampicillin, 10 μg/mL; levofloxacin, 20 μg/mL; vancomycin, 40 μg/mL; and gentamicin, 100 μg/mL.

### 4.3. Susceptibility of Mutants to Antibiotics

In order to assess the effects of *purN* knockout on persister formation, overnight cultures of the relevant *S. aureus* were diluted 1:1000 with TSB in bacterial culture tubes and cultured at 37 °C with shaking (180 rpm). At 5, 9, and 18 h of incubation, cultures were collected, and ampicillin (10 μg/mL), levofloxacin (20 μg/mL), vancomycin (40 μg/mL), and gentamicin (100 μg/mL) were added to assess persister survival. The cultures exposed to drugs were incubated without shaking at 37 °C for up to ten days. Aliquots of cultures exposed to antibiotics were taken at different time points and washed in TSB, and the number of viable cells was counted after serial dilutions.

### 4.4. Construction of Gene Knockout and Overexpression Strains

To construct *purN* knockout mutants of *S. aureus*, we followed the method described previously [15]. The plasmid, pMX10, was used for gene knockout in *S. aureus*. Q5 Master Mix PCR (New England BioLabs) was used for all PCR experiments, and restriction enzymes and T4 DNA Ligase (Thermo Fisher Scientific, Waltham, MA, USA) were used to construct the recombinant plasmids used in this study. The Primers used for *purN* of *S. aureus* gene knockout included purN-uf, purN-ur, purN-df, and purN-dr, and the primer sequences are listed in Supplementary Table S3.

To construct knockout mutants, upstream and downstream fragments of each gene were amplified with the corresponding primers using the genomic DNA of the *S. aureus* wild-type strain Newman as a template. Two fragments of each gene were then used as templates to amplify a fusion fragment with appropriate primers. The fusion fragment and pMX10 plasmid were digested with *Kpn* I and *Mlu* I, respectively, and ligated with T4 DNA ligase, and the recombinant plasmids were transformed into *E. coli* DC10B competent cells. The transformed DC10B was screened on LB agar plates containing ampicillin (100 μg/mL), and the positive clones were verified by restriction digestion and DNA sequencing. The recombinant plasmid was electrotransformed into the *S. aureus* Newman strain, as we described previously [32]. Mutants selection was carried out following the previously published protocol [61]. Using the same method, we also obtained *gltB* knockout mutants of *S. aureus.*

The pRAB11 plasmid was used for inducible overexpression of *purN* in *S. aureus*. The full sequence of *purN* of wild-type *S. aureus* Newman was amplified with the primers OEpurN-f and OEpurN-r (Supplementary Table S3). After digestion with *Kpn*I and *EcoR*I, the fragment was inserted into pRAB11. The recombinant plasmid pRAB11-purN was transformed into *E. coli* DC10B competent cells. The recombinant plasmid pRAB11-purN, was verified by DNA sequencing and then electrotransformed into Δ*purN* and **Δ***gltB* mutants and Newman wild-type to obtain ∆*purN*::pRABpurN, Δ*gltB*::pRBpurN and Newman::pRBpurN, while the empty pRAB11 was transformed into ∆*purN*, Δ*gltB* mutants and Newman wild-type and ∆*purN*::pRAB11, Δ*gltB*::pRAB11 and Newman::pRAB11 were obtained.

### 4.5. RT-qPCR Detected Genes Expression

After the cultures of *S. aureus* were treated with lysostaphin (Shanghai Hi-tech Bioengineering Co., Ltd., Shanghai, China), total RNA was extracted using the Sangon RNeasy kit (Sangon Biotech, China), and the quality and concentration of the extracted RNA were analyzed using a NanoDrop spectrophotometer (Thermo Fisher Scientific, Wilmington, DE, USA). Reverse transcription was performed with SuperScript III First-Strand synthesis (Takara Bio, Japanese) using 1 μg of total RNA that was isolated according to the manufacturer’s instructions. RT-qPCR was performed using SYBR Green Supermix (Yeasen Biotech, Shanghai, China), and the relative fold changes in gene expression were calculated using 16S rRNA as an endogenous control gene [62]. The data represent the results from three independent experiments. The primers for each gene were designed using Primer Premier 5.0 software (PREMIER Biosoft International, San Francisco, USA), and the primer sequences are listed in Supplementary Table S3. All data were analyzed with GraphPad Prism 8.0 (GraphPad Software, San Diego, CA, USA) and compared using the independent-samples *t*-test. Differences with *p*-value < 0.05 were considered statistically significant.

### 4.6. Hemolysis Assay

*S. aureus* Newman::pRAB11, ∆*purN*::pRAB11, ∆*purN*::pRABpurN, and Newman::pRBpurN were inoculated on TSA plates containing 10% sheep blood and Atc (100 ng/mL), incubated at 37 °C for 10, 14, 24 and 48 h, and the hemolysis that formed around the colonies were observed. The hemolysis analysis was conducted as described previously [63] with some modifications. Briefly, Newman::pRAB11, ∆*purN*::pRAB11, ∆*purN*::pRABpurN, and Newman::pRBpurN were cultured in TSB medium with chloramphenicol (10 µg/mL) for 18 h at 180 rpm, diluted 1:1000 and cultured in 5 mL of TSB containing Atc (100 ng/mL) for 10, 14, 24 and 48 h. Each culture was centrifuged at 9000× *g* for 3 min. Then, 200 μL of supernatant was mixed with an equal volume of 4% (*v*/*v*) sheep red blood cells suspended in PBS buffer and incubated at 37 °C for 1.5 h with shaking at 180 rpm. Supernatants were collected after centrifugation (12,000× *g* for 1 min), and the optical density at 540 nm was measured with a spectrophotometer. All experiments were performed in triplicate.

### 4.7. Establishment of an In Vitro S. aureus Biofilm Model

The ability of the *S. aureus* strains to form biofilm was tested in a 96-well plate according to a previously published method [64]. Two hundred microliters of TSB with 0.25% glucose and Atc (100 ng/mL) were transferred to each of the wells on the microtiter plate. Two microliters of each overnight culture of *S. aureus* were transferred to the wells, except for the blank control. Each *S. aureus* strain was tested in three parallel wells. The 96-well plate was incubated at 37 °C for 24 h. The wells were then washed three times with 200 µL of PBS and left to dry at 60 °C for 60 min. Then, 200 µL of crystal violet (0.5% solution, Sigma Aldrich, St. Louis, MO, USA) was added and incubated at room temperature for 30 min. The wells were washed five times with 200 µL of tap water. In order to extract the crystal violet from the biofilm, 200 µL of 33% glacial acetic acid was added. The optical density of the solutions at 550 nm was measured.

### 4.8. Median Lethal Dose Determination

Seventy-five female BALB/c mice weighing approximately 18–22 g were randomly divided into 15 groups to measure the median lethal dose (LD_50_) of the *S. aureus* Newman wild-type strain and the ∆*purN* mutant. The overnight *S. aureus* Newman wild-type and the Δ*purN* were diluted 1:100 in 100 mL TSB and shaken overnight at 37 °C. The cultures were centrifuged at 12,000 rpm for 3 min, and the pellets were washed twice with sterile PBS. After the removal of the supernatant, the pellets were resuspended in 10 mL PBS, and the viable bacteria in the suspension were counted by serial dilution. Then, the suspensions were diluted to form 7 concentration gradients using a double dilution method. Each mouse in each group was injected intraperitoneally with 0.6 mL of a bacterial suspension at doses ranging from 10^8^–10^10^ CFU/mL. After 5 days of observation, the LD_50_ value of each strain was calculated by the Reed-Muench method [65].

### 4.9. Transcriptome Analysis

To identify the key genes regulating the differential responses between the parental Newman strain and Δ*purN* mutant, triplicate samples cultured for 5 h in TSB after dilution of 1:1000 were collected and subjected to high-throughput mRNA transcriptome sequencing. Total RNA was extracted as mentioned above. Sequencing libraries were generated according to the manufacturer’s protocol (NEBNext^®^UltraTM RNA Library Prep Kit for Illumina^®^) [66]. Cleaved RNA fragments were copied into first-strand cDNA using reverse transcriptase and random primers. The cDNA library preferentially selected fragments of 200–250 bp in length, which were prepared by the AMPure XP system. Then, the fragment products were amplified by Illumina cBot and sequenced on an Illumina HiSeq 2500 system (Illumina, San Diego, CA USA). Library construction and sequencing were performed at the Shanghai Applied Protein Technology Co., Ltd. By using Hisat2 (v2.0.5) (https://daehwankimlab.github.io/hisat2/manual/ (accessed on 19 August 2020)), paired-end clean reads were aligned to the reference genome of *S. aureus* Newman on the NCBI website. The number of reads corresponding to each gene was calculated using Feature Counts v1.5.0-p3. Then, each gene fragment per kilobase million (FPKM) was calculated based on the gene lengths and read counts mapped to this gene. In order to control the false discovery rate, Benjamini and Hochberg’s approach was used to adjust the *p*-values to compare FPKM values between the mutant and wild-type groups. Genes with *P*_adj_ < 0.05 and log_2_ fold change >2 or <−2 were defined as differentially expressed genes (DEGs). RT-qPCR, which was performed in triplicate, was used to confirm the RNA expression levels, and the primer sequences are listed in Supplementary Table S2.

### 4.10. Protein-Protein Interaction Network

In order to explore the interactive relationships among DEGs, the web portal for the STRING database (http://www.string-db.org/ (accessed on 1 April 2021)) was used for protein–protein interaction (PPI) network analysis. The following two criteria were applied to detect the important nodes: (1) medium confidence equal to 0.4 and (2) network clustering by K-means clustering.

## Figures and Tables

**Table 1:** Bacteria and plasmids used in this study.

Strains or Plasmid	Relevant Genotype and Property	Source or Reference
*S. aureus* Strains		
Newman	Clinical isolate, ATCC 25904	ATCC
Δ*purN*	Newman with a deletion of *purN*	This study
Newman::pRAB11	Newman with pRAB11	This study
∆*purN*::pRAB11	∆*purN* with pRAB11	This study
∆*purN*::pRBpurN ∆*gltB*::pRAB11 ∆*gltB*::pRBpurN	∆*purN* with pRAB11-purN ∆*gltB* with pRAB11 ∆*gltB* with pRAB11-purN	This study This study This study
*Escherichia coli* strains		
DC10B	∆*dcm* in the DH10B background; Dam methylation only	[33]
plasmids		
pMX10	A pKOR1 derivate for gene knockout, Cm^R^, Amp^R^	[29]
pRAB11	Atc inducible shuttle plasmid, Cm^R^, Amp^R^	[15]
pRAB11-purN	Overexpression plasmid for *purN*	This study

Cm^R^: Chloramphenicol resistance; Amp^R^: Ampicillin resistance. The antibiotics were used at the following concentrations: ampicillin at 100 μg/mL and chloramphenicol at 10 μg/mL to maintain the plasmids resistance.

## Data Availability

Not applicable.

## Supplementary Materials

The following supporting information can be downloaded at: https://www.mdpi.com/article/10.3390/antibiotics11121702/s1, Table S1: DEGs between Δ*purN* and its parental strain (log_2_ fold change greater than 2 or less than −2); Table S2: Oligonucleotide sequences of RT-qPCR primers used in this study. RT-qPCR verified DEGs between Δ*purN* and its parental strain from transcriptome analysis. Results were normalized using 16S rRNA and expressed as fold change (mean ± SD, *p* < 0.05); Table S3: Primers and oligonucleotides used in this study; Figure S1: The growth curves for *S. aureus* Newman::pRAB11, ∆*purN*::pRAB11, ∆*purN*::pRABpurN, ∆*gltB*::pRAB11, ∆*gltB*::pRABpurN and Newman::pRABpurN strains; Figure S2: Drug exposure results of 18-h culture of Newman::pRAB11, ∆*purN*::pRAB11, ∆*purN*::pRABpurN, and Newman::pRBpurN to ampicillin (**A**), vancomycin (**B**), gentamicin (**C**) and levofloxacin (**D**); Figure S3: Drug exposure results of 18-h culture of Newman::pRAB11, ∆*gltB*::pRAB11, ∆*gltB*::pRABpurN, and Newman::pRBpurN to ampicillin (**A**), levofloxacin (**B**), gentamicin (**C**) and vancomycin (**D**).
