# Supplementary material for: PurN Is Involved in Antibiotic Tolerance and Virulence in Staphylococcus aureus
Source: Antibiotics (Basel). 2022 Nov 25;11(12):1702. doi: 10.3390/antibiotics11121702 (PMC9774800; doi:10.3390/antibiotics11121702)
Supplement: Supplementary file 1 [file antibiotics-11-01702-s001.zip › antibiotics-2005503-supplementary.pdf]

Supplementary Table S1. DEGs between Δ*purN* and its parental strain (log_2 fold change greater than 2 or less than -2)

Gene name	Log_2 fold change	FDR*	Description
purN	-9.83	1.5949E-21	phosphoribosylglycinamide formyltransferase
lukS	-7.47	8.2716E-98	leukocidin/hemolysin toxin subunit S
hlgC	-7.26	0	gamma-hemolysin component C
NWMN_0676	-6.44	0	hypothetical protein
lukF	-6.22	0	leukocidin/hemolysin toxin subunit F
hlgB	-6.20	0	gamma hemolysin, component B
NWMN_0677	-6.08	0	hypothetical protein
hla	-5.59	1.1798E-248	alpha-hemolysin precursor
sbi	-5.51	0	immunoglobulin G-binding protein Sbi
NWMN_1066	-5.32	1.7066E-160	hypothetical protein
saeR	-5.18	0	DNA-binding response regulator SaeR
NWMN_2321	-5.09	2.8266E-20	hypothetical protein
NWMN_1070	-5.0	9.0232E-262	hypothetical protein
NWMN_1905	-4.82	0.020249	hypothetical protein
NWMN_0402	-4.79	1.5088E-305	hypothetical protein
NWMN_1069	-4.79	4.6575E-165	hypothetical protein
NWMN_1873	-4.78	7.16E-14	truncated beta-hemolysin
saeS	-4.74	0	sensor histidine kinase SaeS
hlgA	-4.58	2.02E-296	gamma-hemolysin component A
scn	-4.26	0	complement inhibitor SCIN
NWMN_2398	-3.90	4.5E-71	C-terminal part of fibronectin binding protein A
fnbA	-3.84	2.29E-276	fibronectin binding protein A precursor
NWMN_2396	-3.76	1.03E-223	C-terminal part of fibronectin binding protein B
map	-3.66	0	MHC class II analog protein
chp	-3.64	3.11E-16	chemotaxis-inhibiting protein CHIPS
NWMN_1084	-3.62	2.09E-161	anti protein
NWMN_0760	-3.61	1.59E-89	thermonuclease precursor
coa	-3.51	2.78E-118	staphylocoagulase precursor
splA	-3.29	0.00093681	serine protease SplA
thrB	-3.25	9.22E-97	homoserine kinase
geh	-3.17	7.3E-99	truncated triacylglycerol lipase precursor
NWMN_0362	-3.15	7.27E-43	hypothetical protein
thrS	-3.11	1.53E-249	threonyl-tRNA synthetase
fnbB	-3.10	1.76E-91	fibronectin binding protein B precursor
gltD	-2.91	4.28E-91	glutamate synthase subunit beta
set4nm	-2.90	5.19E-33	superantigen-like protein
NWMN_0757	-2.76	8.28E-11	secreted von Willebrand factor-binding protein precursor
feoB	-2.72	1.62E-135	ferrous iron transport protein B

hisG	-2.71	0.00029184	ATP phosphoribosyltransferase catalytic subunit
gltB	-2.62	1.43E-108	glutamate synthase, large subunit
cobW	-2.61	1.15E-32	cobalamin synthesis protein
NWMN_0165	-2.60	2.04E-20	hypothetical protein
thrC	-2.56	5.42E-165	threonine synthase
NWMN_1067	-2.43	8.55E-19	formyl peptide receptor-like 1 inhibitory protein
ald	-2.39	2.4E-125	alanine dehydrogenase
NWMN_1881	-2.38	0.0011256	phage amidase
NWMN_0759	-2.36	1.64E-18	hypothetical protein
ilvA	-2.30	2.9E-155	threonine dehydratase
set11nm	-2.26	0.001068	superantigen-like protein
ilvD	-2.26	4.69E-37	dihydroxy-acid dehydratase
NWMN_1347	-2.24	3.79E-119	amino acid permease
NWMN_0071	-2.21	1.55E-60	acetoin reductase
leuC	-2.17	7.77E-61	isopropylmalate isomerase large subunit
splB	-2.15	0.0028652	serine protease SplB
thrA	-2.07	4.16E-30	aspartate kinase
bioW	-2.06	4.85E-18	6-carboxyhexanoate--CoA ligase
set9nm	-2.03	2.19E-05	superantigen-like protein
NWMN_2449	-2.0	2.42E-11	hypothetical protein
NWMN_2332	2.03	1.15E-226	hypothetical protein
NWMN_0485	2.07	2.67E-67	UvrB/UvrC motif-containing protein
argR	2.09	3.32E-10	arginine repressor
prsA	2.11	3.02E-30	peptidyl-prolyl cis/trans-isomerase
grpE	2.13	1.76E-119	heat shock protein GrpE
NWMN_2223	2.23	2.68E-35	hypothetical protein
NWMN_1721	2.24	2.69E-07	hypothetical protein
hrcA	2.31	8.74E-91	heat-inducible transcription repressor
pstS	2.32	6.00E-10	phosphate ABC transporter phosphate-binding protein PstS
NWMN_2510	2.33	1.71E-157	glycine betaine aldehyde dehydrogenase
NWMN_0486	2.35	2.64E-235	ATP:guanido phosphotransferase
NWMN_0845	2.39	4.75E-199	ATP-dependent Clp protease, ATP-binding subunit ClpB
NWMN_0230	2.39	0.023216	hypothetical protein
NWMN_2468	2.58	0.012911	acetyltransferase, GNAT family protein
NWMN_2266	2.65	2.89E-31	hypothetical protein
NWMN_1834	3.15	2.43E-15	hypothetical protein
NWMN_0537	3.18	5.65E-167	hypothetical protein
NWMN_0542	3.53	7.79E-204	hypothetical protein
NWMN_1639	3.56	9.10E-07	hypothetical protein
NWMN_2265	4.24	6.42E-123	hypothetical protein
NWMN_2261	4.70	0	ABC transporter ATP-binding protein

NWMN_2304	4.75	0.020218	hypothetical protein
NWMN_2262	5.13	0	hypothetical protein
NWMN_0232	5.18	0.0044976	hypothetical protein

*: *P* values were adjusted using the false discovery rate (FDR) method.

Supplementary Table S2. Oligonucleotide sequences of RT–qPCR primers used in this study. RT–qPCR verified DEGs between Δ*purN* and its parental strain from transcriptome analysis. Results were normalized using 16S rRNA and expressed as fold change (mean±SD, *P*<0.05).

Genes	Forward	Reverse	Fold change
saeS	5′- GCGATGAAGGTATTGGCA-3′	5′-AATCCAGAACCACCCGTT-3′	2.98±1.21
saeR	5′-CCATTTAGTCCAAGGGAACTC-3′	5′-TCACGGTATTAGCATCTTCG-3′	6.98±0.97
ilvA	5′- GCTTGTTGTTTCGCTTGTGG-3′	5′- AAGGCATTATCGCAGCATCT-3′	1.86±0.64
NWMN_1873	5′- ATGATGGTGAAAAAAACAA-3′	5′- ATACAAAACGGTCGATAAC-3′	6.55±1.12
lukS	5′- TTTGAATGAATTGAGCCT-3′	5′- ATATTGGGAAAAACGGTA-3′	3.55±1.32
hla	5′- ATTGGTAGTCATCACGA-3′	5′- AGCAGATAACTTCCTTG-3′	5.89±3.11
hlgC	5′- TTATCTGTGAGCTTACTTGC-3′	5′- TTTTATCTTCTGTCCTTTTG-3′	3.42±2.18
lukF	5′- TCAGACACAGTTACAGGCA-3′	5′- GTTGGAAAGTAGAAGCACA-3′	4.92±0.72
NWMN_2510	5′- ACCAGACTCAAACGCACGTC-3′	5′- AGTGGGTTGAAAGCGCGAAT-3′	0.16±0.11
NWMN_2262	5′- ACACAACAACAACGTGATGAGCT -3′	5′- AACGGTGCTTGCTCTGCTTG-3′	0.14±0.53
NWMN_2266	5′- TGGTTACTCATAGGCGTCGTCT -3′	5′- TGTTAGTGGAAACCAACCGGC-3′	0.73±1.12
NWMN_0485	5′- TCGTCCGCAGAGTTCAAGGT-3′	5′-TGGCTGCTTCCTCAAAATCTTGG-3′	0.56±0.83
NWMN_0845	5′-TTCGGCATTAGAGCGTCGTTTCC -3′	5′- ATTCAGCGGCAGCAACTAAGGC-3′	0.48±0.67
16S rRNA	5′-CGTGCTACAATGGACAATACAAA-3′	5′- ATCTACGATTACTAGCGATTCCA-3′	

Supplementary Table S3. Primers and oligonucleotides used in this study

Primer	Sequence (5′ → 3′)	Source or reference
16S-f	CGTGCTACAATGGACAATACAAA	[62]
16S-r	ATCTACGATTACTAGCGATTCCA	[62]
purN-f	GCTGGCTACATGCGTCTAAT	This study
purN-r	TCCCGTATCCATACCACTATCA	This study
purN-uf	GGGGTACCGACGCGCCATATAGTGGCTTATG	This study
purN-ur	TAGCTTTCTTCATCACATACATACTCCTGTTATACC CCCAACAATTCAAT	This study
purN-df	ATTGAATTGTTGGGGGTATAACAGGAGTATGTATG TGATGAAGAAAGCTA	This study
purN-dr	CGACGCGTATCTTGAACTAAATATCCGCCAGAT	This study
OEpurN-f	GGGGTACCGGTCATATTGTGAAAAATGAGT	This study
OEpurN-r	CGGAATTCTACCTGTTTTATTTGATACGCT	This study
hla-f	ATTGGTAGTCATCACGA	This study
hla-r	AGCAGATAACTTCCTTG	This study
hlgA-f	AGGTGTTAAATGGGGAGT	This study
hlgA-r	TCTGGGACGAAATAGTCT	This study
hlgB-f	AATGTTGGCTGGGGAGT	This study

hlgB-r	CGCTATGAAGTTTTGGC	This study
hlgC-f	TTATCTGTGAGCTTACTTGC	This study
hlgC-r	TTTTATCTTCTGTCCTTTTG	This study
lukS-f	TTTGAATGAATTGAGCCT	This study
lukS-r	ATATTGGGAAAAACGGTA	This study
lukF-f	TCAGACACAGTTACAGGCA	This study
lukF-r	GTTGGAAAGTAGAAGCACA	This study
eta-f	TCCAAATCAAGAAAGCAAA	This study
eta-r	TGAGACTTCAAAAAGCACC	This study
sea-f	TGATAACCATAATAAGCAC	This study
sea-r	AAACTGAAAATAAAGAGAG	This study
coa-f	AGTTAAAATTCCACAGGG	This study
coa-r	TTCTAAAATAGGGTTCGT	This study

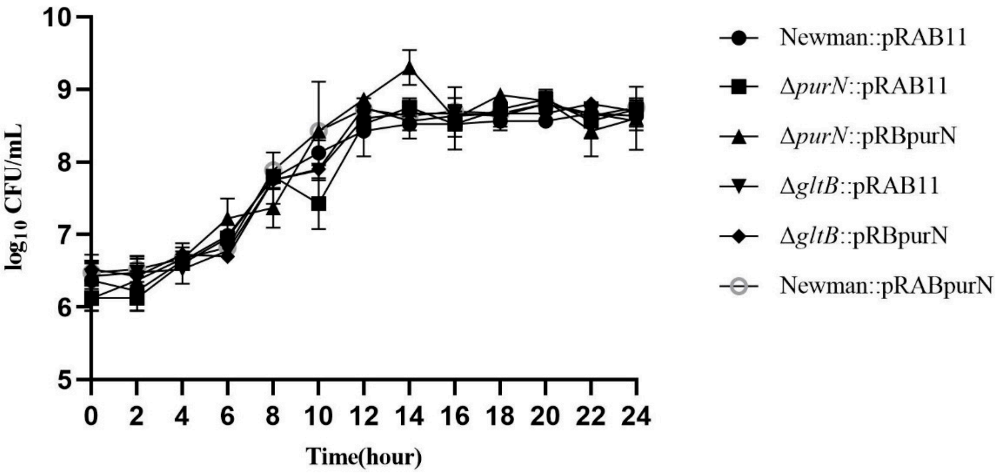

Supplementary Figure S1. The growth curves for *S. aureus* Newman::pRAB11, Δ*purN*::pRAB11, Δ*purN*::pRABpurN, Δ*gltB*::pRAB11, Δ*gltB*::pRABpurN and Newman::pRABpurN strains.

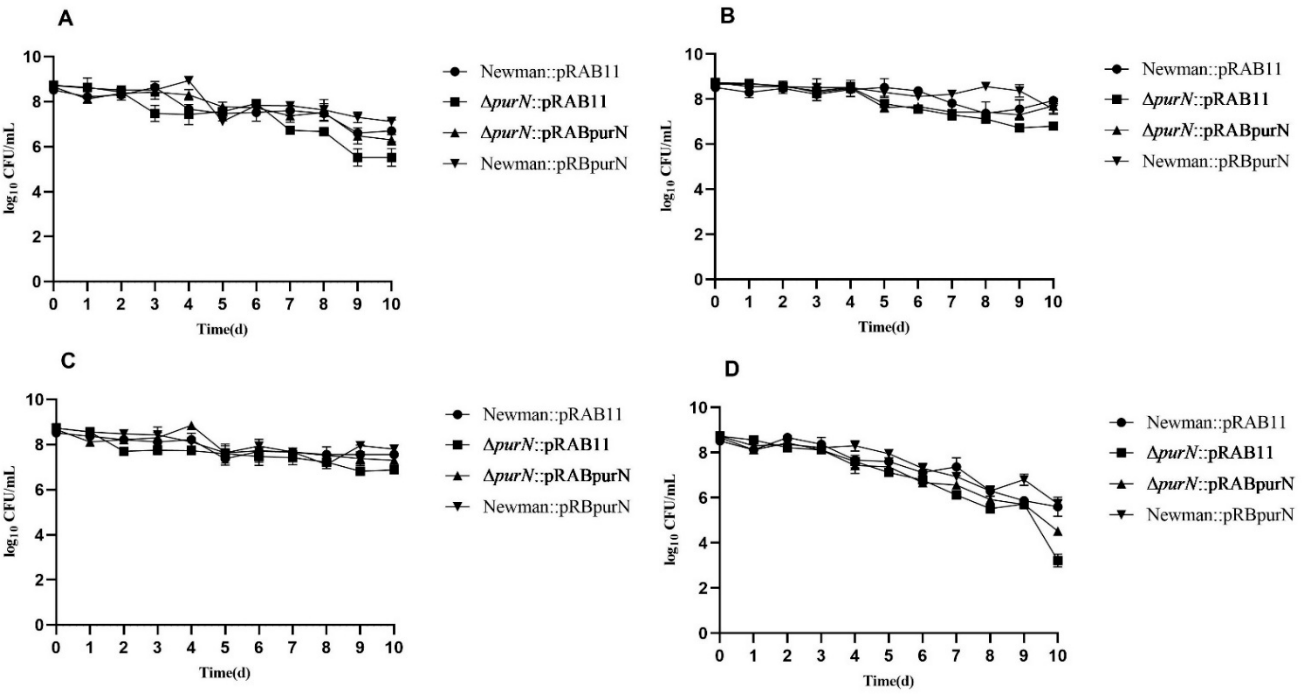

Supplementary Figure S2. Drug exposure results of 18-hour culture of Newman::pRAB11, Δ*purN*::pRAB11, Δ*purN*::pRABpurN and Newman::pRBpurN to ampicillin (A), vancomycin (B), gentamicin (C) and levofloxacin (D) .

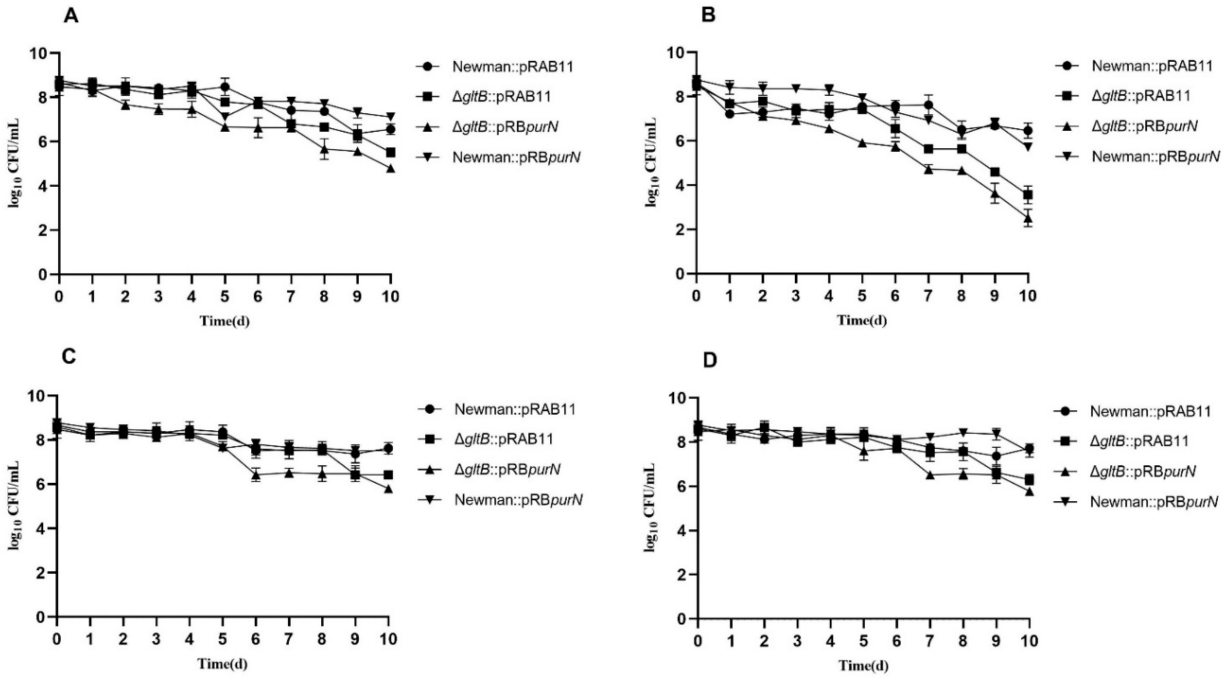

Supplementary Figure S3. Drug exposure results of 18-hour culture of Newman::pRAB11, Δ*gltB*::pRAB11, Δ*gltB*::pRABpurN and Newman::pRBpurN to ampicillin (A), levofloxacin(B), gentamicin (C) and vancomycin (D).
